# The Impact of the COVID-19 Pandemic on the Mental Health of First-Year Undergraduate Students Studying at a Major Canadian University: A Successive Cohort Study

**DOI:** 10.1177/07067437221094549

**Published:** 2022-04-21

**Authors:** Nathan King, William Pickett, Daniel Rivera, Jin Byun, Melanie Li, Simone Cunningham, Anne Duffy

**Affiliations:** 1 Department of Public Health Sciences, 4257 Queen’s University, Kingston, ON, Canada; 2 Department of Health Sciences, 7497 Brock University, St. Catharines, ON, Canada; 3 Department of Pharmacology and Toxicology, 7938 University of Toronto, ON, Canada; 4 Faculty of Health Sciences, Queen’s University, Kingston, ON, Canada; 5 Biology Department, Queen’s University, Kingston, ON, Canada; 6 Department of Biomedical and Molecular Sciences, Queen’s University, Kingston, ON, Canada; 7 Department of Psychiatry, Division of Student Mental Health, Queen’s University, Kingston, ON; 8 Department of Psychiatry, 6396 University of Oxford, Oxford, UK

**Keywords:** university student, mental health, well-being, COVID-19, post-secondary, anxiety, depression, substance use, self-harm

## Abstract

**Objective:**

To examine the impact of the COVID-19 pandemic on first year undergraduate student mental health.

**Methods:**

As part of the Queen’s University *U-Flourish Student Well-Being and Academic Success* study, three successive cohorts of students entering undergraduate studies in 2018 (pre-pandemic), 2019 (transitional), and 2020 (during pandemic) completed electronic surveys at entry and completion of first year. Validated self-report measures were used to assess mental health status including symptom levels of anxiety, depression, and insomnia, self-harm and frequency of substance use. Propensity matching and multivariable log-binomial regression were used in comparisons of mental health indicators across the cohorts.

**Results:**

Clinically significant symptoms of depression, anxiety, insomnia, and self-harm were reported more frequently in the 2020–2021 cohort, coincident with remote learning and pandemic restrictions. In female students, screen positive rates for anxiety and depression, and suicidal ideation increased from about one-third to just under one-half in association with the pandemic (χ^2^, *p* < .01), while increases in mental health concerns were less pronounced among males. Among females, increases in clinically significant symptoms over first year appeared greatest during the pandemic year, while striking decreases in alcohol consumption in both females and males were reported in that same year. Studying under pandemic conditions had a negative impact on student well-being, social relationships and school connectedness, quality of learning experience, leisure activities, and optimism about future prospects.

**Conclusions:**

Mental health concerns including anxiety, depression and sleep problems increased in first year students during the pandemic, especially among females, while alcohol use declined. These findings highlight the negative mental health impact associated with studying under pandemic restrictions involving remote learning and social distancing.

## Abrègè

**Objectif:**

Examiner l’effet de la pandémie de la COVID-19 sur la santé mentale d’étudiants de première année du premier cycle.

**Méthode:**

Dans le cadre de l’étude longitudinale sur le bien-être et la réussite scolaire des étudiants universitaires U-Flourish, de l’Université Queen’s, trois cohortes successives d’étudiants commençant leurs études de premier cycle en 2018 (pré-pandémie), 2019 (transitoire), et 2020 (durant la pandémie) ont rempli des sondages électroniques au début et à la fin de la première année. Des mesures d’auto-évaluation validées ont servi à évaluer l’état de la santé mentale notamment les niveaux des symptômes d’anxiété, de dépression, et d’insomnie, d’autodestruction ainsi que la fréquence de l’utilisation de substances. Les scores de propension et la régression log-binomiale multivariée ont été utilisés dans les comparaisons des indicateurs de santé mentale dans les cohortes.

**Résultats:**

Les symptômes de dépression, d’anxiété, d’insomnie et d’autodestruction cliniquement significatifs ont été rapportés plus fréquemment dans la cohorte de 2020-2021, ce qui coïncide avec l’apprentissage à distance et les restrictions pandémiques. Chez les étudiantes de sexe féminin, les taux de dépistage positifs d’anxiété et de dépression, et l’idéation suicidaire passaient d’environ un tiers à presque une demie en association avec la pandémie (χ^2^, p < 0,01), alors que les accroissements des préoccupations de santé mentale étaient moins prononcés chez le sexe masculin. Chez les femmes, les augmentations des symptômes cliniquement significatifs dans la première année apparaissaient plus grandes durant l’année de la pandémie, alors que des diminutions frappantes de la consommation d’alcool chez les femmes et les hommes ont été déclarées dans cette même année. Étudier dans les conditions de la pandémie avait un effet négatif sur le bien-être des étudiants, les relations sociales et la connexion à l’école, la qualité de l’expérience d’apprentissage, les activités de loisirs et l’optimisme quant aux débouchés futurs.

**Conclusions:**

Les problèmes de santé mentale, notamment l’anxiété, la dépression et les problèmes de sommeil ont augmenté chez les étudiants de première année durant la pandémie, particulièrement chez les femmes, alors que l’alcool a été moins consommé. Ces résultats soulignent l’effet négatif sur la santé mentale associé à l’étude dans des restrictions pandémiques comportant l’apprentissage à distance et la distanciation sociale.

## Introduction

Entry to university marks an important developmental period for young people. At this stage, most students are tasked with leaving home, making new friends, being more autonomous, navigating new learning environments and health care services, and acquiring study skills to meet higher education standards.^1^ This key period for psychological and sociological development coincides with a time of accelerated brain development^2^ and the peak period of risk for the onset of major mental disorders.^3^ Further, university students have to navigate important lifestyle decisions (i.e., drug and alcohol use, exercise and sleep regulation) in the face of varying abilities to cope in healthy ways, which contributes to their overall well-being. It is therefore not surprising that universities in Canada,^4,5^ as in other countries,^6^ continue to experience a high demand for student mental health support.

Restrictions related to the COVID-19 pandemic posed additional challenges for university students.^7^ During its first and second waves, Canadian university campuses were physically closed to most students, and teaching methods shifted to online formats.^8,9^ In-person socialization was suspended, and remote learning which included asynchronous pre-recorded lectures increased the demand on students for self-regulation and time management. Many students lost a degree of autonomy as they were required to study from home, sometimes under suboptimal circumstances (e.g., due to lack of study space, and family/housemate relationships).^7^ However, there are only sparse data to describe the impact of these pandemic-related changes on the mental health of university students studying in Canada.

The *U-Flourish Student Well-Being and Academic Success study* (*U-Flourish*) was launched at Queen’s University in 2018.^10^ Since its inception, all undergraduate students entering first year (including those in professional schools) have been invited to complete a biannual survey that focuses on mental health and well-being, and related risk and protective factors.

The existence of *U-Flourish* provides a unique opportunity to examine the mental health of undergraduate students over the first year of university before and after the pandemic was declared. Analysis of the experiences of successive student cohorts can assist with the planning of mental health support tailored to student need and increase understanding of the important contributing factors. Our study objectives were as follows: to examine clinically significant levels of anxiety, depression, insomnia, self-harm, and suicidality, and substance use at school entry (i) and compare changes in these mental health outcomes over the first academic year (ii) in students studying under pandemic compared to pre-pandemic conditions, and (iii) to explore the impact of studying during the pandemic from the student perspective.

We hypothesized that while clinically significant symptoms of common mental health concerns would continue to be highly prevalent over time, screen positive rates at school entry and over the first year would be increased in students studying under pandemic conditions, while substance use and other behaviours traditionally related to socializing in-person would remain unchanged or become less prevalent.

## Methods

### Setting

Queen’s University is in Kingston, Ontario, Canada; a small city of approximately 500,000 located between the major centres of Toronto, Montreal, and Ottawa. Queen’s is a medium-sized, publicly funded university that accepts approximately 4,500 undergraduate students annually. In the fall of 2019, 90% of first-year students were 18 years or younger on the first day of school and 73% attended high school in Ontario.^11^ Prior to pandemic related restrictions, more than 90% of first-year students lived in residence on campus.^12^

### Data Source

As described elsewhere, first year undergraduate students at Queen’s University were invited to complete an online mental health survey at the start of the fall term (September) and end of the spring term prior to final exams (in March) as part of the U-Flourish study.^10^ Three successive cohorts of students entering university for the academic years of 2018–2019 (Cohort 1; pre-pandemic), 2019–2020 (Cohort 2; transitional), and 2020–2021 (Cohort 3; during pandemic) contributed. Validated measures were used to assess mental health outcomes, with supplemental information about the student experience gathered via free text responses. All student participants read a letter of information and provided informed consent prior to completing the survey.^10^

Through a student-designed and led engagement campaign, almost 60% of eligible students completed the baseline fall survey in 2018^4^; a response rate replicated in 2019. In 2020, the response rate for the baseline survey dropped to 23%, at a time when the university had shifted to an online format and in-person engagement activities were not possible. Response rates to the follow-up survey in successive cohorts were 64% in the 2018 cohort, then dropped to 37% and 37% in 2019 and 2020, respectively.

The COVID-19 pandemic was officially declared in Canada in March of 2020.^13^ Hence, students enrolled in the 2019–2020 cohort were exposed to the restrictions related to the pandemic including campus closure and online proctored examinations only during the very end of their academic year, while the 2020–2021 cohort studied under pandemic conditions for their entire academic year. This meant that the campus was closed, and most curriculum was offered remotely through a mixture of online synchronous and asynchronous formats and examinations were remotely proctored.

### Study Variables

Measures of mental health, well-being, and associated risk and protective factors considered in the U-Flourish survey have been described in detail elsewhere.^4,10^ A brief synopsis follows.

#### Demographics

Students reported their age in years, gender, international/domestic student status, ethnicity, and the highest level of education completed by their parents. Individual programs of study were obtained from the university administrative database.

#### Diagnosed Mental Illness

Students reported whether they (personal history) and any of their first-degree relatives (family history) had ever been diagnosed with any of the following mental disorders: mood, anxiety, psychotic, eating, sleep, neurodevelopmental, and substance use.

#### Childhood exposures to risk

The survey included questions from the Childhood Experience of Care and Abuse Questionnaire (CECA)^14^ to ascertain a self-reported history of physical abuse, sexual abuse, and bullying by peers during childhood.

#### Mental Health Treatment

Students indicated whether they were currently receiving treatment or support for a mental health concern, and in Cohorts 2 and 3 students also reported on their lifetime history of mental health treatment at baseline.

#### Symptoms of Common Mental Illnesses

Current symptoms of depression were measured using the 9-item Patient Health Questionnaire (PHQ-9).^15^ Responses to the items were summed (range = 0–27), with a score of ≥ 10 indicating a “screen positive” or clinically significant depressive symptoms.^15^ Current symptoms of anxiety were measured using the Generalized Anxiety Disorder 7-item scale (GAD-7).^16^ Responses to the items were summed (range = 0–21), with a score of ≥ 10 indicating a screen positive or clinically significant anxiety symptoms.^16^ Lifetime history of self-harm, suicide ideation, and suicide attempts were measured using questions from the Columbia Suicide Rating Scale.^17^ Sleep problems were assessed using the 8-item Sleep Condition Indicator (SCI-8).^18^ Responses to the items were summed with a score of ≤ 16 out of 32 indicating reduced sleep quality and clinically significant symptoms of insomnia.^18^ Person-mean imputation was used to calculate scale scores if one item was missing.

#### Substance Use

Past month frequency of alcohol consumption, binge drinking (5 + alcoholic drinks on one occasion) and cannabis use (“weekly or more” vs. “less than weekly”) was reported. The number of drinks containing alcohol consumed on a typical day when the student was drinking was grouped as “5 or more” versus “less than 5.” Illicit drug use in the past month, was based on the use of any of the following: cocaine (coke, crack, etc.), other street drugs (e.g., opioids, LSD, speed, MDMA, ecstasy), or a prescription drug without a prescription or to get “high, buzzed or numbed out.”

#### COVID-19 Impact (Cohort 3; 2020–2021)

Eleven items assessing the perceived impact of the pandemic and associated social distancing and remote learning, developed in collaboration with student partners, were added to the follow-up survey for Cohort 3. Students rated the impact on their university experience, including perceptions of remote learning, social relationships, leisure activities, and finances on a 5-point scale from 1 = “Very negative” to 5 = “Very positive.”^19^ In addition, in both the baseline and follow-up surveys, students in Cohort 3 provided free text responses to the open-ended question “*Are there any other significant impacts related to the COVID-19 pandemic on your mental health, wellbeing, or education that you would like to comment on?*”

### Analysis

All analyses were conducted using SAS Version 9.4 (*SAS* Institute, Cary NC).

*Objective 1. Comparison of mental health at university entry*: The proportion of students screening positive for anxiety, depression, insomnia, self-harm and suicidal thoughts and attempts was compared between the three successive cohorts at school entry. To account for differences between the cohorts in risk profiles that may have resulted from the lower response rate during the pandemic, a subset of 1,330 students from Cohorts 1 (2018–2019) and 2 (2019–2020) were identified by 1:1 matching to Cohort 3 (2020–2021) on gender, then further on propensity scores. This score was developed using plausible risk factors for mental disorders: age, international status, personal, and family history of a mental disorder, childhood physical and/or sexual abuse, childhood bullying, and parental education. The baseline samples for Cohorts 1, 2, and 3 were 3,029, 2,949, and 1,472, respectively; after restricting to students with complete data on variables used in the matching procedure the samples were 2,509, 2,669, and 1,330.

Prevalence levels and associated 95% confidence intervals for the mental health outcomes reported at school entry were described in each propensity score-matched cohort. All analyses were stratified by male or female gender; reports from non-binary students were excluded due to small cell sizes and associated privacy concerns. Significant differences in prevalence between cohorts were estimated via chi-square tests. In a planned sensitivity analysis, we examined relationships between cohort membership and mental health outcomes using all available data. Relative Risks were estimated via multivariable log-binomial regression models, with adjustment for the variables included in the propensity score-matched analysis.

*Objective 2. Changes in mental health over the first year under pandemic (2020–2021) compared to pre-pandemic (2018–2019) conditions:* The analysis was limited to students in Cohort 1 and Cohort 3 who completed both the baseline and follow-up surveys because these cohorts provide a clear comparison of students studying prior to versus during the pandemic. Absolute differences in the prevalence of mental health outcomes at follow-up compared to baseline were described. Multivariable log-binomial regression was used to estimate the relative risks of each outcome over the academic year associated with cohort membership. Models were stratified by gender, and further adjusted for age, international status, lifetime and family history of mental illness, childhood physical or sexual abuse, childhood bullying, parental education, and baseline status of the mental health outcome of interest. Models examining risk of self-harm, suicide ideation, and suicide attempts over the academic year were adjusted for lifetime history at baseline. After restricting to students with complete covariate data the longitudinal samples were 1,549 for Cohort 1 and 402 for Cohort 3. In both cohorts, students lost to follow-up reported similar mental health at baseline (symptoms of anxiety, depression, and insomnia) as those who completed the follow-up (χ^2^, *p *≥ .30). These analyses were 80% powered to detect relative risks of 1.22 to 2.78 in females and 1.51 to 4.00 in males (α = 0.05, two-sided).

*Objective 3. Student experience of the impact of the pandemic (2020–2021; Cohort 3): Quantitative.* We described the proportion of students reporting a “Negative; 1-2,” “Positive; 4-5,” or “Neutral; 3” impact on each of the impact items, included on the follow-up survey. *Qualitative.* Three student investigators were supervised in using the framework technique,^20^ to identify common themes in an iterative process of reviewing text responses to the open-ended impact question. Briefly, the student researchers read through the responses and individually assigned “labels” to each response which corresponded to identifiable themes. Following this initial coding, they convened to discuss and develop an agreed upon list of themes. Further discussion yielded a consensus final set of key themes.

### Ethics

The U-Flourish study followed the ethical principles set-out in the Declaration of Helsinki and was approved for ethical compliance by the Queen’s University and Affiliated Teaching Hospitals Research Ethics Board (HSREB PSIY-609-18).

## Results

Characteristics of the propensity-matched samples used to compare mental health outcomes at university entry are presented in Table 1. The matched cohorts were similar with respect to all key variables examined. Specifically, at entry to university student participants were mainly between 18 and 19 years old, two-thirds were female, and most were domestic students of White or Asian ethnicity, who had parents with higher education backgrounds. The leading programs of study were the Arts, Humanities and Social Sciences, followed by Life and Physical Sciences, and Engineering. About one in four students reported a lifetime history of a diagnosed mental disorder and 40% reported an immediate family member with a lifetime mental disorder. Specifically, at university entry, approximately one in seven students reported a lifetime history of a mood disorder (13.2–15.4% across cohorts). In terms of major risk factors, about one in five students reported physical or sexual abuse during childhood. Further, over one-fifth of students entering university reported a lifetime history of treatment for a mental health concern; and the rate of current mental health treatment at university entry was: 10.8%, 9.3%, and 14.1% across Cohorts 1–3, respectively.

**Table 1.** Description of the Propensity Matched Samples of First Year Undergraduate Students at University Entry Across Successive Cohorts.

	Cohort 1 Fall 2018		Cohort 2 Fall 2019		Cohort 3 Fall 2020	
	*n*	(%)	*n*	(%)	*n*	(%)
Total	1330	(100)	1330	(100)	1330	(100)
Age, *Mean (SD)*^a^	18.6	(2.4)	18.4	(1.4)	18.8	(2.6)
Gender^b^						
Female	894	(67.2)	894	(67.2)	894	(67.2)
Male	413	(31.1)	413	(31.1)	413	(31.1)
Other identity	23	(1.7)	23	(1.7)	23	(1.7)
International student, *Yes*^a^	153	(11.5)	167	(12.6)	146	(11.0)
Ethnicity						
White	894	(67.3)	856	(64.4)	791	(59.5)
Asian	260	(19.6)	260	(19.6)	320	(24.1)
Black	16	(1.2)	20	(1.5)	24	(1.8)
Indigenous	3	(0.2)	5	(0.4)	5	(0.4)
Other	14	(1.1)	35	(2.6)	46	(3.5)
Multiple	142	(10.7)	153	(11.5)	144	(10.8)
*Missing*	*1*	* *	*1*	* *	* *	* *
Program of study						
Arts, humanities, and social sciences	425	(32.0)	506	(38.1)	576	(43.3)
Life and physical sciences	392	(29.5)	316	(23.8)	278	(20.9)
Engineering and applied science	193	(14.5)	231	(17.4)	215	(16.2)
Business	162	(12.2)	149	(11.2)	90	(6.8)
Computing	42	(3.2)	31	(2.3)	49	(3.7)
Nursing	37	(2.8)	50	(3.8)	40	(3.0)
Medicine	41	(3.1)	14	(1.1)	30	(2.3)
Law	38	(2.9)	33	(2.5)	52	(3.9)
Lifetime history of mental disorder, *Yes*^a^	380	(28.6)	354	(26.6)	393	(29.6)
Childhood physical or sexual abuse, *Yes*^a^	268	(20.2)	283	(21.3)	295	(22.2)
Childhood bullying, *Yes*^a^	270	(20.3)	278	(20.9)	275	(20.7)
Parental education, highest completed^a^						
Degree in professional school or doctorate	269	(20.2)	259	(19.5)	274	(20.6)
Master’s degree	254	(19.1)	266	(20.0)	271	(20.4)
Bachelor’s degree or trades/apprenticeship	607	(45.6)	596	(44.8)	591	(44.4)
Completed high school or less	200	(15.0)	209	(15.7)	194	(14.6)
Family history of mental disorder, *Yes*^a^	574	(43.2)	575	(43.2)	548	(41.2)

*Note:* (1)^a^ indicates a variable that was included in the propensity score matching; (2)^b^ cohorts exact matched on gender.

**Figure 1.**
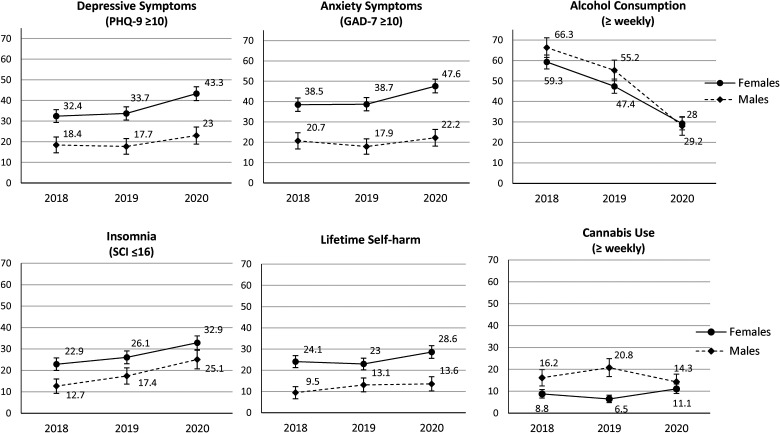
Prevalence (95% confidence interval) of clinically significant depressive and anxiety symptoms, insomnia, lifetime self-harm, alcohol consumption, and cannabis use at school entry across successive cohorts, in female and male students.

*Objective 1. Comparison of mental health at university entry*: Table 2 and accompanying Figure 1 describe the prevalence of mental health outcomes reported at entry to university within each of the three matched cohorts. Symptoms meeting threshold cut-offs for depression, anxiety, insomnia, and lifetime self-harm all were reported more frequently in Cohort 3 (Fall 2020), coincident with studying under pandemic restrictions. Specifically, in female students screen positive rates for anxiety, depression, and suicidal ideation increased from about one-third to just under one-half from Cohorts 1 (Fall 2018) and 2 (Fall 2019) to Cohort 3. Similarly, in male students screen positive rates for anxiety, depression, and insomnia shifted from under one-fifth to around one-quarter during the pandemic and suicidal ideation increased from about one-quarter to one-third. In contrast, there was a striking decrease in alcohol consumption in Cohort 3 in both females and males, while the regular use of cannabis did not vary substantially by survey cycle. Specifically, binge drinking rates weekly or greater halved from 24% to 12% in females (χ^2^, *p* < .001) and from 45% to 20% in males (χ^2^, *p* < .001) under pandemic conditions compared to the year prior. These findings were consistent with those from the sensitivity analysis that included all available data (Supplemental Table 2).

**Table 2.** Screen Positive Rates of Reported Mental Health Symptoms and Substance use in Successive Propensity Matched Samples of First Year Undergraduate Students at Entry to University Before (Fall 2018 and Fall 2019) and During (Fall 2020) the Pandemic (%), by Gender.

	Females				Males			
Mental health indicators	*n*	2018	2019	2020	*n*	2018	2019	2020
Depressive symptoms (PHQ-9 ≥ 10)	855	32.4^a^	33.7^a^	43.3^b^	396	18.4	17.7	23.0
Anxiety symptoms (GAD-7 ≥ 10)	857	38.5^a^	38.7^a^	47.6^b^	396	20.7	17.9	22.2
Insomnia (SCI ≤ 16)	821	22.9^a^	26.1^a^	32.9^b^	379	12.7^a^	17.4^a^	25.1^b^
Lifetime self-harm	880	24.1^a^	23.0^a^	28.6^b^	411	9.5	13.1	13.6
Lifetime suicidal ideation	882	32.8^a^	38.4^b^	49.5^c^	410	25.1^a^	28.5^a^	36.3^b^
Lifetime suicide attempt	881	9.5	8.7	9.4	411	4.1	5.1	4.6
Substance use								
Alcohol consumption, ≥ weekly	810	59.3^a^	47.4^b^	29.2^c^	371	66.3^a^	55.2^b^	28.0^c^
Binge drinking, ≥ weekly	822	/	23.9^a^	12.5^b^	380	/	45.5^a^	20.3^b^
Typical drinking day, 5 + drinks	825	/	27.0^a^	15.3^b^	380	/	55.3^a^	30.5^b^
Illicit drug use in the past month	820	/	7.9	6.0	379	/	14.8 ^a^	6.9^b^
Cannabis use, ≥ weekly	819	8.8	6.5^a^	11.1^b^	378	16.2	20.8 ^a^	14.3^b^

*Note:* (1) chi-square test for difference in proportions, (2) different superscripts (a, b, c) indicate significantly different proportions for the mental health indicator (χ^2^, *p* < .05), (3) for depressive and anxiety symptoms, and insomnia the values represent the proportion meeting the cut-off for clinically significant symptoms, (4) each cohort was propensity score matched using: age, international student status, personal and family history of a mental disorder, childhood physical and/or sexual abuse, childhood bullying, and parental education, (5) the typical drinking day and binge drinking items were not available in the 2018 cohort.

*Objective 2. Changes in mental health over the first year under pandemic compared to pre-pandemic conditions*: Table 3 describes the relative risk of reporting the various mental health indicators at follow-up in Cohort 3 (2020–2021; pandemic conditions) versus at follow-up in Cohort 1 (2018–2019; pre-pandemic conditions), adjusting for differences in mental health status at baseline. In both cohorts, clinically significant symptoms of common mental health concerns were higher upon follow-up when compared to baseline levels. Among females, after controlling for baseline mental health status, those in Cohort 3 appeared to be at increased risk of reporting symptoms meeting cut-off thresholds over the study year compared with members of Cohort 1, although only the effect for anxiety reached statistical significance. Among male students, there were no statistically significant differences between cohorts in screen positive rates for anxiety, depression or insomnia at follow-up.

**Table 3.** Changes in the Prevalence of Mental Health and Substance use Outcomes in Undergraduate Students Over the First Year of University in Cohort 1 (2018–2019; Pre-Pandemic) and Cohort 3 (2020–2021; During Pandemic), and Risk of Mental Health and Substance Use Outcomes at the end of First Year in Cohort 3 Compared to Cohort 1.

	Absolute change in % at follow-up				Risk of outcome at follow-up		
Females	2018–2019		2020–2021		2018–2019	2020–2021	
**Mental health indicators**	**Diff**	**(95% CI)**	**Diff**	**(95% CI)**	**RR**	^a^ **RR**	**(95% CI)**
Depressive symptoms (PHQ-9 ≥ 10)^b^	9.3	(5.4–13.2)	10.5	(2.4–18.7)	1.00	1.18	(0.97–1.43)
Anxiety Symptoms (GAD-7 ≥ 10)^b^	5.8	(1.8–9.9)	10.3	(2.2–18.5)	1.00	1.22	(1.02–1.46)
Insomnia (SCI ≤ 16)^b^	8.5	(5.0–12.1)	9.9	(1.8–18.0)	1.00	1.15	(0.92–1.44)
Self-harm, *past 6 months*					1.00	1.42	(0.94–2.15)
Suicidal ideation, *past 6 months*					1.00	1.22	(0.91–1.62)
Suicide attempt, *past 6 months*					1.00	0.68	(0.23–2.06)
**Substance use**							
Alcohol consumption, ≥ weekly	−0.9	(−5.0–3.2)	0.9	(−6.1–7.9)	1.00	0.67	(0.51–0.88)
Cannabis use, ≥ weekly	5.6	(3.2–8.0)	4.7	(−0.2–9.6)	1.00	0.85	(0.56–1.27)
**Males**							
**Mental health indicators**							
Depressive symptoms (PHQ-9 ≥ 10)^b^	10.6	(4.8–16.4)	11.4	(−0.3–23.2)	1.00	1.03	(0.72–1.49)
Anxiety symptoms (GAD-7 ≥ 10)^b^	9.1	(3.3–14.8)	7.7	(−3.6–19.0)	1.00	1.03	(0.70–1.52)
Insomnia (SCI ≤ 16)^b^	10.2	(5.3–15.0)	8.5	(−3.5–20.4)	1.00	1.19	(0.79–1.79)
Self-harm, *past 6 months*					1.00	1.38	(0.55–3.49)
Suicidal ideation, *past 6 months*					1.00	1.24	(0.73–2.10)
Suicide attempt, *past 6 months*					1.00	2.83	(0.64–12.5)
**Substance use**							
Alcohol consumption, ≥ weekly	4.4	(−2.5–11.2)	1.7	(−9.0–12.4)	1.00	0.63	(0.40–0.98)
Cannabis use, ≥ weekly	5.4	(0.3–10.6)	1.8	(−6.9–10.5)	1.00	0.70	(0.39–1.27)

*Notes:* (1)^a^ adjusted for age, international/domestic status, childhood physical or sexual abuse, childhood bullying, parental education, personal and family history of a mental disorder, and baseline status of the given indicator (self-harm and suicidality were adjusted for lifetime history), (2) ^b^ met threshold for clinically significant symptoms, (3) sample sizes vary by indicator (2018 Cohort Ranges = 1105–1142 females, 389–407 males; 2020 Cohort Ranges = 263–285 females, 104–117 males), (4) binge drinking, typical number of drinks, and illicit drug use items could not be compared between cohorts.

Although not statistically significant, students in Cohort 3 appeared to be at increased risk of self-harm and suicide ideation over the academic year (Table 3). Self-harm over the past 6 months was reported by 6.7% of female and 3.9% of male students in Cohort 1 compared to 11.9% and 6.1% in Cohort 3. Suicide ideation increased from 14.8% to 25.2% in females, and from 12.8% to 17.4% in males. Cohort membership was not significantly associated with risk of a suicide attempt, which was reported by a small proportion of students in both cohorts. In students studying under pre-pandemic conditions, 18 (1.6%) females and 6 (1.5%) males reported having made a suicide attempt over the past 6 months, compared to 4 (1.4%) females and 4 (3.5%) males studying under pandemic conditions. Finally, contrary to the previous findings, during the pandemic both male and female students were significantly less likely to report weekly alcohol consumption at follow-up. There was also a non-significant decrease in cannabis use at follow-up in the pandemic compared to pre-pandemic cohort, in both males and females.

**Figure 2.**
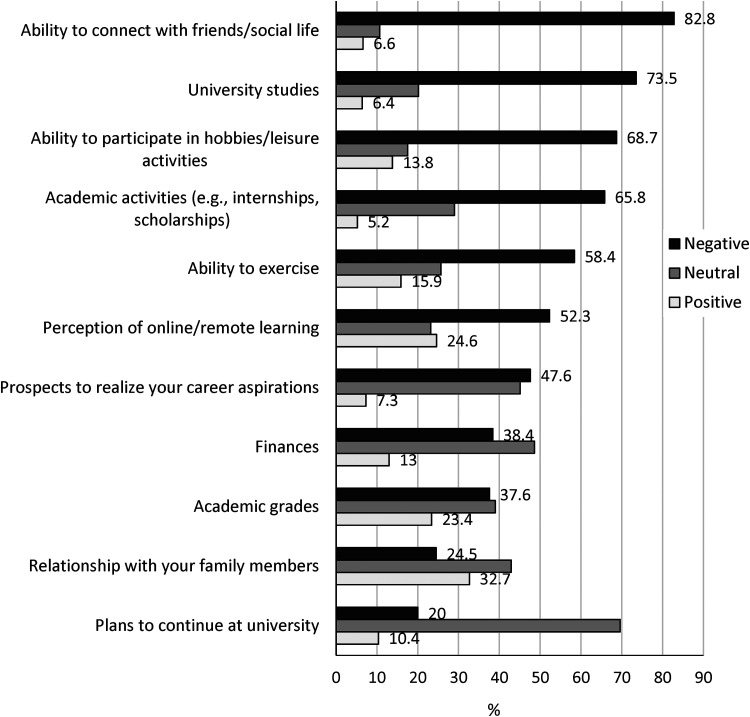
Impact of the COVID-19 pandemic and associated social distancing and remote learning on your...

*Objective 3. Student experience of the impact of the pandemic (2020–2021; Cohort 3):* The perceived impact of the pandemic and associated social distancing and remote learning reported by students in Cohort 3 (2020–2021) is presented in Figure 2. Nearly three-quarters (73.5%) of students reported that the pandemic had a negative impact on their university studies, and over half (52.3%) reported a negative perception of online/remote learning. Significant proportions of students also reported a negative impact on activities important to mental health and coping including their ability to exercise (58.4%), participate in hobbies or leisure activities (68.7%), and connect with friends and their social life (82.8%).

Following these items, students had the opportunity to voice any other specific impacts of the pandemic on their mental health, wellbeing, or education. Major themes identified from the open-text responses (*n* = 451) included: (1) mental health concerns, (2) reduced social connectedness and university belonging, (3) academic and learning concerns, (4) physical health and lifestyle concerns, and (5) financial and job prospect concerns.

## Discussion

We compared three successive cohorts of first-year undergraduate students to examine the impact of the COVID-19 pandemic on student mental health at entry to university and over the first academic year. The main findings included a sustained high level of clinically significant symptoms of common mental health concerns at entry to university, which were elevated in students studying under pandemic conditions. Specifically, screen-positive levels of self-reported depressive, anxiety, and insomnia symptoms, and self-harm were significantly higher among students who entered university during the pandemic compared to those entering the year prior. Observed increases were greatest amongst females, nearly half of whom met screening thresholds for at least one mental health outcome upon entry to university during the pandemic. While screen-positives increased over the academic year before and during the pandemic, there appeared to be a greater increase in female students studying under pandemic conditions, particularly for symptoms of anxiety. Conversely, reported levels of problematic substance use (alcohol binging and/or regular recreational drug use) were lower during the pandemic, consistent with reductions in alcohol consumption reported among young adults in Canada,^21,22^ associated with decreases in social gatherings and COVID-related restrictions.^21^ Students indicated that the pandemic had negatively impacted their lives and university studies and shared concerns around reduced social and school connectedness, the quality of their learning experience and specifically remote learning, as well as concerns about future academic and career prospects.

The potential impacts of the COVID-19 pandemic on student mental health have been reported in Canada^23,24^ and globally, although many of these studies are limited in sample size and scope of enquiry, and the findings vary by educational setting and geographic context. At Carleton University the pandemic has had a negative effect on undergraduate student stress levels and mental health, especially among females.^23^ Copeland et al.^25^ reported modest increases in externalizing symptoms and attention problems at the onset of the pandemic in students at the University of Vermont (UVM), with evidence of a protective effect of having been enrolled in a Wellness program; while internalizing symptoms remained stable. In a broader sample of US college students, Conrad et al.^9^ documented increases in symptoms of grief, loneliness, and generalized anxiety with the onset of the pandemic, which were especially pronounced in students required to vacate campus residences and other forms of university housing. Increases in anxiety and stress were documented in first-year French students during the pandemic lockdown, but particularly amongst those who did not relocate to live with family.^26^ In a study of Swiss undergraduates, levels of anxiety, depression, stress, and loneliness increased during the pandemic.^27^ In an exploratory analysis, social isolation and lack of emotional support were associated with worse mental health outcomes. Evans et al.^28^ compared 254 UK undergraduate psychology students in pre-pandemic versus “lockdown” conditions, and documented significant increases in some symptoms (e.g., depression), reductions in others (wellbeing, alcohol use), and no change in symptoms of anxiety, loneliness, or sleep quality. Toth et al.^29^ reported findings more consistent with our own, with elevated levels of depression and anxiety in pandemic versus non-pandemic student samples. Similarly, Canadian students with no pre-existing mental health problems reported increases in psychological distress during the pandemic.^24^ Finally, a major study of medical students in China reported that up to one-quarter of participants experienced mild to severe symptoms of anxiety associated with worries about academic delays and financial issues.^30^

A significant proportion of students in our study reported that the pandemic and associated restrictions had negatively impacted their social life and ability to connect with friends, their university studies, including concerns about the quality of online learning and their grades, their ability to participate in exercise and leisure activities, and their future prospects and finances. In keeping with these quantitative findings, students shared that the pandemic and associated restrictions had negatively impacted their mental health through heightened feelings of stress, anxiety, and hopelessness. Difficulty with time management, blurred work-life boundaries, perceived lower quality of online learning, and inadequate academic support were commonly expressed academic challenges. Pandemic-related public health restrictions resulted in students feeling socially isolated from friends and disconnected from the campus community. General concerns about the future, including job prospects and whether a “return to normal” would occur, were common sources of distress. Established wellness, coping, and lifestyle-enriching activities were disrupted due to the reduction of available recreational and meeting spaces, as well as the emphasis on physical distancing. The emergence of novel or worsening eating, sleep, and substance use problems were described and, in some cases, considered to be a direct effect of COVID-19-related changes.

*Strengths and Limitations*. Strengths of the *U-Flourish* survey study include the large representative first-year samples and the embedded longitudinal component. The matched samples were broadly representative according to age, ethnicity, program of study, and international status,^11^ and we achieved high response rates across cycles compared to other North American surveys of post-secondary students.^31^ Study recruitment was aided by a purposeful student-led engagement campaign, which also afforded opportunity for targeted knowledge exchange. The survey itself included validated screening measures and subscales which remained largely consistent across study waves and allowed us to assess mental outcomes under pandemic and non-pandemic conditions. Furthermore, open-ended survey responses allowed us to hear the student voice about studying under pandemic conditions. However, limitations of this analysis warrant comment. Self-report measures are validated screening measures but are not diagnostic. Measures of substance use (i.e., illicit drug use) are subject to social desirability bias and may be underreported as a result. During the pandemic, the survey response rate significantly dropped, raising the potential for selection bias. We were however able to adjust for differences in risk profiles between the cohorts using multiple statistical approaches, but owing to differences in response rates and attrition some selection bias may remain. Finally, not all substance use items were available across all time points.

Study findings have several important clinical implications. First, we found evidence of a significant degree of adversity experienced by first-year undergraduate students related to the pandemic, and a substantial negative impact on student mental health. While this finding is not surprising given the emerging literature, it underscores the scope of need in terms of planning for university student mental health support. Reported increases in clinically significant levels of mental health problems will inevitably increase the already high student demand for support. Moreover, while reports of some mental health concerns (e.g., recreational drug use, alcohol misuse) declined, this trend is expected to be temporary as it had more to do with the lack of opportunity to socialize with peers. Therefore, a student-tailored and accessible clinical triage system with therapeutic benefit from the first contact and that maps student need to the appropriate level of support seems a priority.^5^

Future studies are needed to examine the underlying associations and mechanisms driving mental health outcomes and to assess sustained effects of the pandemic on the mental health burden and academic experiences of postsecondary students. Given the sustained and increasing need for student mental health support, optimization of models guiding the provision of services is required in collaboration with students, along with testing in accordance with evidence-based principles. For example, both universal health promotion and targeted interventions organized in an integrated stepped care model have been proposed, but as yet not rigorously studied.^5,32^

## Conclusion

We found evidence that the COVID-19 pandemic was associated with a significant increase in mental health concerns in first year undergraduate students, especially among females. Students studying under pandemic conditions expressed feeling isolated from peers, a lack of membership with the university, reduced quality of their educational experience, limited recreational outlets and future academic and career uncertainty. These findings address the identified need for large-scale longitudinal data to inform the mental health burden in undergraduate students and underscore the substantial need in terms of university student mental health support. Future research on the sustained effects of the pandemic and underlying mechanisms is needed to inform evidence-based prevention and early intervention efforts.

## Supplemental Material

sj-pdf-1-cpa-10.1177_07067437221094549 - Supplemental material for The Impact of the COVID-19 Pandemic on the Mental Health of First-Year Undergraduate Students Studying at a Major Canadian University: A Successive Cohort Study Supplemental material, sj-pdf-1-cpa-10.1177_07067437221094549 for The Impact of the COVID-19 Pandemic on the Mental Health of First-Year Undergraduate Students Studying at a Major Canadian University: A Successive Cohort Study by Nathan King, PhD, William Pickett, PhD, Daniel Rivera, BSc, Jin Byun, Melanie Li, Simone Cunningham, PhD, and Anne Duffy, MD, FRCPC in The Canadian Journal of Psychiatry
