# Supplementary material for: The Impact of the COVID-19 Pandemic on the Mental Health of First-Year Undergraduate Students Studying at a Major Canadian University: A Successive Cohort Study
Source: Can J Psychiatry. 2022 Apr 21;68(7):499–509. doi: 10.1177/07067437221094549 (PMC9096012; doi:10.1177/07067437221094549)
Supplement: sj-pdf-1-cpa-10.1177_07067437221094549 - Supplemental material for The Impact of the COVID-19 Pandemic on the Mental Health of First-Year Undergraduate Students Studying at a Major Canadian University: A Successive Cohort Study [file sj-pdf-1-cpa-10.1177_07067437221094549.pdf]

SUPPLEMENTAL MATERIALS

Supplementary Table 1. Description of the longitudinal samples used to examine changes in mental health and substance use over the first year of university

	Cohort 1 2018/19		Cohort 2 2019/20		Cohort 3 2020/21	
	n	(%)	n	(%)	n	(%)
Total	1570	(100)	939	(100)	404	(100)
Age, *Mean(SD)*	18.1	(1.5)	18.0	(1.0)	19.0	(3.0)
Gender						
Female	1144	(72.9)	693	(73.8)	286	(70.8)
Male	407	(25.9)	239	(25.5)	117	(29.0)
Other Identity	19	(1.2)	7	(0.8)	1	(0.3)
International Student, *Yes*	115	(7.3)	78	(8.3)	39	(9.4)
Ethnicity						
White	1064	(67.9)	594	(63.3)	232	(57.4)
Asian	293	(18.7)	182	(19.4)	94	(23.3)
Black	24	(1.5)	14	(1.5)	9	(2.2)
Indigenous	0	(0.0)	1	(0.1)	2	(0.5)
Other	21	(1.3)	27	(2.9)	15	(3.7)
Multiple	166	(10.6)	121	(12.9)	52	(12.9)
Missing	*2*		*0*		*0*	
Program of Study						
Arts, Humanities, & Social Sciences	504	(32.1)	317	(33.8)	162	(40.1)
Life and Physical Sciences	489	(31.2)	294	(31.3)	75	(18.6)
Engineering and Applied Science	263	(16.8)	158	(16.8)	85	(21.0)
Business	160	(10.2)	92	(9.8)	22	(5.5)
Computing	51	(3.3)	25	(2.7)	11	(2.7)
Nursing	42	(2.7)	30	(3.2)	17	(4.2)
Medicine	37	(2.4)	7	(1.7)	16	(4.0)
Law	24	(1.5)	16	(5.0)	16	(4.0)
Lifetime history of mental disorder, *Yes*	412	(26.2)	282	(30.0)	134	(33.2)
Childhood physical or sexual abuse, *Yes*	237	(15.1)	170	(18.1)	83	(20.5)
Childhood bullying, *Yes*	330	(21.0)	202	(21.5)	85	(21.0)
Parental Education, Highest Completed						
Degree in Professional School or Doctorate	364	(23.2)	218	(23.2)	95	(23.5)
Master's	375	(23.9)	205	(21.8)	82	(20.3)
Bachelors Degree or trades/apprenticeship	644	(41.0)	424	(45.2)	174	(43.1)
Completed High School or less	187	(11.9)	92	(9.8)	53	(13.1)
Family history of mental disorder, *Yes*	687	(43.8)	434	(46.2)	216	(53.5)

Supplementary Table 2. Results of sensitivity analysis using all available data: Multivariable binomial logistic regression examining the association between year of study (Cohort membership) and indicators of mental health and substance use behaviours of undergraduate students entering university before (Fall 2018 and 2019) and during (Fall 2020) the pandemic, by gender

**FEMALES**	**2018**		**2019**		**2020**		**2018**	**2019**		**2020**	
**Mental Health Indicators**	**n**	**(%)**	**n**	**(%)**	**n**	**(%)**	**RR**	**[a]RR**	**(95% CI)**	**[a]RR**	**(95% CI)**
Depressive Symptoms (PHQ-9≥10)	1692	(30.7)	1789	(35.7)	855	(43.3)	1.00	1.12	(1.00-1.26)	1.33	(1.17-1.53)
Anxiety Symptoms (GAD-7≥10)	1692	(37.8)	1787	(37.7)	857	(47.6)	1.00	0.97	(0.87-1.08)	1.21	(1.07-1.38)
Insomnia (SCI <16)	1685	(20.5)	1754	(27.3)	821	(32.9)	1.00	1.29	(1.12-1.48)	1.50	(1.27-1.76)
Lifetime self-harm	1692	(22.3)	1785	(22.8)	880	(28.6)	1.00	0.95	(0.83-1.10)	1.15	(0.98-1.35)
Lifetime suicidal ideation	1691	(31.9)	1787	(37.7)	882	(49.5)	1.00	1.14	(1.02-1.28)	1.45	(1.27-1.64)
Lifetime suicide attempt	1692	(7.3)	1786	(7.7)	881	(9.4)	1.00	0.98	(0.77-1.26)	1.08	(0.82-1.44)
**Substance Use**											
Alcohol consumption, ≥ weekly	1655	(57.1)	1753	(49.9)	810	(29.2)	1.00	0.86	(0.78-0.94)	0.51	(0.44-0.59)
Binge drinking, ≥ weekly			1760	(26.0)	822	(12.5)		1.00	referent	0.49	(0.39-0.61)
Typical drinking day, 5+ drinks			1760	(28.9)	825	(15.3)		1.00	referent	0.53	(0.43-0.65)
Illicit drug use in the past month			1758	(7.5)	820	(6.0)		1.00	referent	0.75	(0.54-1.05)
Cannabis use, ≥ weekly	1663	(8.4)	1745	(7.6)	819	(11.1)	1.00	0.84	(0.66-1.07)	1.21	(0.93-1.59)
**MALES**	**2018**		**2019**		**2020**		**2018**	**2019**		**2020**	
**Mental Health Indicators**	**n**	**(%)**	**n**	**(%)**	**n**	**(%)**	**RR**	**[a]RR**	**(95% CI)**	**[a]RR**	**(95% CI)**
Depressive Symptoms (PHQ-9≥10)	782	(20.5)	823	(16.3)	396	(23.0)	1.00	0.77	(0.61-0.97)	1.03	(0.79-1.34)
Anxiety Symptoms (GAD-7≥10)	783	(21.2)	824	(17.0)	396	(22.2)	1.00	0.79	(0.63-0.99)	0.96	(0.74-1.25)
Insomnia (SCI <16)	781	(12.6)	788	(16.6)	379	(25.1)	1.00	1.31	(1.01-1.71)	1.83	(1.38-2.43)
Lifetime self-harm	784	(9.2)	822	(11.2)	411	(13.6)	1.00	1.15	(0.84-1.56)	1.26	(0.88-1.79)
Lifetime suicidal ideation	784	(24.4)	822	(25.9)	410	(36.3)	1.00	1.02	(0.84-1.25)	1.38	(1.11-1.71)
Lifetime suicide attempt	784	(4.3)	821	(4.4)	411	(4.6)	1.00	0.94	(0.58-1.50)	0.89	(0.50-1.57)
**Substance Use**											
Alcohol consumption, ≥ weekly	770	(60.9)	792	(55.6)	371	(28.0)	1.00	0.90	(0.79-1.03)	0.46	(0.37-0.57)
Binge drinking, ≥ weekly			799	(46.2)	380	(20.3)		1.00	referent	0.46	(0.36-0.59)
Typical drinking day, 5+ drinks			799	(54.7)	380	(30.5)		1.00	referent	0.58	(0.47-0.71)
Illicit drug use in the past month			796	(13.1)	379	(6.9)		1.00	referent	0.47	(0.30-0.73)
Cannabis use, ≥ weekly	766	(17.8)	791	(20.5)	378	(14.3)	1.00	1.11	(0.88-1.40)	0.81	(0.59-1.11)

Notes: (1) [a] Adjusted for age, international/domestic status, personal or family history of a mental disorder, childhood physical or sexual abuse, childhood bullying, parental education, (2) binge drinking, typical number of drinks, and illicit drug use items could not be compared between all cohorts.

Supplementary Table 3. Description of first year undergraduate students at university entry across successive cohorts; samples used in the sensitivity analysis examining mental health outcomes at school entry using all available data

	**Cohort 1 Fall 2018**		**Cohort 2 Fall 2019**		**Cohort 3 Fall 2020**	
	n	(%)	n	(%)	n	(%)
Total	2509	(100)	2666	(100)	1330	(100)
Age, *Mean(SD)*	18.2	(2.0)	18.0	(1.2)	18.8	(2.6)
Gender						
Female	1695	(67.6)	1804	(67.7)	894	(67.2)
Male	786	(31.3)	834	(31.3)	413	(31.1)
Other Identity	28	(1.1)	28	(1.1)	23	(1.7)
International Student, *Yes*	236	(9.4)	238	(8.9)	146	(11.0)
Ethnicity						
White	1692	(67.5)	1776	(66.7)	791	(59.5)
Asian	489	(19.5)	443	(16.6)	320	(24.1)
Black	35	(1.4)	37	(1.4)	24	(1.8)
Indigenous	4	(0.2)	7	(0.3)	5	(0.4)
Other	35	(1.4)	84	(3.2)	46	(3.5)
Multiple	252	(10.1)	317	(11.9)	144	(10.8)
Missing	*2*		*2*			
Program of Study						
Arts, Humanities, & Social Sciences	866	(34.5)	997	(37.4)	576	(43.3)
Life and Physical Sciences	720	(28.7)	747	(28.0)	278	(20.9)
Engineering and Applied Science	394	(15.7)	460	(17.3)	215	(16.2)
Business	285	(11.4)	269	(10.1)	90	(6.8)
Computing	88	(3.5)	56	(2.1)	49	(3.7)
Nursing	60	(2.4)	88	(3.3)	40	(3.0)
Medicine	52	(2.1)	14	(1.3)	30	(2.3)
Law	44	(1.8)	35	(3.2)	52	(3.9)
Lifetime history of mental disorder, *Yes*	583	(23.2)	710	(26.6)	393	(29.6)
Childhood physical or sexual abuse, *Yes*	404	(16.1)	488	(18.3)	295	(22.2)
Childhood bullying, *Yes*	541	(21.6)	527	(19.8)	275	(20.7)
Parental Education, Highest Completed						
Degree in Professional School or Doctorate	598	(23.8)	640	(24.0)	274	(20.6)
Master's Degree	605	(24.1)	590	(22.1)	271	(20.4)
Bachelor's Degree or trades/apprenticeship	977	(38.9)	1154	(43.3)	591	(44.4)
Completed High School or less	329	(13.1)	282	(10.6)	194	(14.6)
Family history of mental disorder, *Yes*	906	(36.1)	1030	(38.6)	548	(41.2)

Supplementary Table 4. Themes identified from open-text responses to the question: "*Are there any other significant impacts related to the COVID-19 pandemic on your mental health, wellbeing, or education that you would like to comment on?*"

Theme	Representative Quotes
Mental health concerns	"Covid-19 has made me feel more pessimistic, anxious and upset"<br>"I have been struggling with maintaining a strong mental health"<br>"The COVID-19 pandemic makes me hopeless that life will never go back to normal"
Reduced social connectedness and university belonging	"I feel less connected to others and more inclined to think negatively."<br>"Being isolated and having limited contact with friends makes me lonely"
Academic and learning concerns	"Online classes have become stressful and overwhelming. I cannot seem to keep up with the workload."<br>"Since COVID-19 has pushed everything online my grades have dropped. I don't do well with online learning."<br>"I don't feel I'm getting a proper education"
Physical health and lifestyle concerns	"I feel overworked, and as if I don't even have time to maintain healthy physical habits such as exercising."<br>"It is more difficult to maintain wellness activities"<br>"Lockdown triggered an extreme laziness and irregular sleep pattern"
Financial and job prospects concerns	"I can't work because of COVID and am constantly worrying about how I'll pay my university fees"<br>"Learning from home is difficult motivation wise, and financially harsh when considering the lack of physical resources in conjunction with job difficulties."
Neutral or positive outlooks or experiences*	"COVID-19 has not significantly impacted my lifestyle other than my safety precautions when leaving and returning home. "<br>"I really like online courses, which allow me to manage my time more properly and have more time staying with my family."

Notes: (1) Themes are based on 451 free-text responses (Fall 2020, n=358; Spring 2021, n=93), (2) *The proportion of students reporting neutral or positive outlooks or experiences was relatively low
